# Physicochemical Characteristics of Chitosan-Based Hydrogels Modified with *Equisetum arvense* L. (Horsetail) Extract in View of Their Usefulness as Innovative Dressing Materials

**DOI:** 10.3390/ma14247533

**Published:** 2021-12-08

**Authors:** Magdalena Głąb, Anna Drabczyk, Sonia Kudłacik-Kramarczyk, Marcel Krzan, Bożena Tyliszczak

**Affiliations:** 1 Department of Materials Science, Faculty of Materials Engineering and Physics, Cracow University of Technology, 37 Jana Pawła II Av., 31-864 Krakow, Poland; bozena.tyliszczak@pk.edu.pl; 2 Jerzy Haber Institute of Catalysis and Surface Chemistry, Polish Academy of Sciences, 8 Niezapominajek St., 30-239 Krakow, Poland; marcel.krzan@ikifp.edu.pl

**Keywords:** hydrogel dressings, *Equisetum arvense* L. (horsetail) extract, sorption capacity, wettability, percentage elongation

## Abstract

This work focused on obtaining and characterizing hydrogels with their potential application as dressing materials for chronic wounds. The research included synthesizing chitosan-based hydrogels modified with *Equisetum arvense* L. (horsetail) extract via photopolymerization, and their characteristics determined with regard to the impact of both the modifier and the amount of crosslinker on their properties. The investigations included determining their sorption properties and tensile strength, evaluating their behavior in simulated physiological liquids, and characterizing their wettability and surface morphology. The release profile of horsetail extract from polymer matrices in acidic and alkaline environments was also verified. It was proved that hydrogels showed swelling ability while the modified hydrogels swelled slightly more. Hydrogels showed hydrophilic nature (all contact angles were <77°). Materials containing horsetail extract exhibited bigger elasticity than unmodified polymers (even by 30%). It was proved that the extract release was twice as effective in an acidic medium. Due to the possibility of preparation of hydrogels with specific mechanical properties (depending on both the modifier and the amount of crosslinker used), wound exudate sorption ability, and possibility of the release of active substance, hydrogels show a great application potential as dressing materials.

## 1. Introduction

In recent years, hydrogel dressings used in the treatment of chronic wounds [1], burns [2], and other skin injuries such as bedsores [3] or serious skin abrasions [4] have been of great interest. Such dressings consist of natural polymers and/or synthetic polymers crosslinked physically or chemically [5,6]. They mostly consist of water, with a water content of up to 90%. Importantly, the water environment provides a much greater degree of regeneration, supporting cell proliferation in the rebuilding epidermis. Therefore, these materials are considered to form an appropriate environment by accelerating the epithelization process [7,8]. Hydrogel dressings are a barrier to microorganisms and, importantly, show very good sorption properties because they are able to easily absorb large quantities of wound exudates [9].

The dressing materials, based on synthetic polymers, were described by Lin et al. The developed hydrogels were prepared via a solvent casting method. The main component of these materials was a synthetic and hydrophilic polymer, poly(vinyl alcohol) (PVA), while co-doped hydroxyapatite (CoHA) was used as a modifying agent. It was demonstrated that prepared composite hydrogels showed no cytotoxic properties. Furthermore, it was also proved that hydrogels containing CoHA were characterized by better mechanical properties and biological activity compared with unmodified PVA-based polymers [10]. Next, poly(acrylic acid)-based hydrogel foils showing good bioadhesive properties were presented. In order to enrich these materials with antibacterial properties, they were additionally modified with metronidazole, i.e., an antibiotic with protozoal and bactericidal activity against anaerobic microorganisms. The prepared hydrogels showed no cytotoxic properties and were characterized by high bactericidal activity against *Escherichia coli*, *Staphylococcus aureus*, and *Streptococcus mutans* [11]. Hydrogel dressings for burn wounds also constitute a main research topic of Jackson et al. Their work aimed at developing dressing materials with prolonged antimicrobial activity, and that could be obtained with low financial outlay. PVA was used as a base of the dressing, while silver nitrate was applied as a modifying agent providing antibacterial properties [12]. In turn, Chen et al. characterized dressing materials based on natural polymers. Silk fibroin- and chitosan-based hydrogels were obtained by direct current electrodeposition process. Studies on the properties of these materials showed that they positively affect the HEK-93 cell proliferation and antibacterial activity against *Escherichia coli* and *Staphylococcus aureus*. Furthermore, in vivo histological studies confirmed that prepared hydrogels promoted the wound re-epithelization process and, importantly, enhanced granulation tissue formation [13]. Hydrogel dressings based on natural polymers were also investigated by Amante et al. [14], Wang et al. [15] and Dioniz et al. [16]. Conversely, materials comprised of a combination of natural and synthetic polymers were also studied [17,18].

One of the main, and most important, characteristics of hydrogel materials is the possibility of their easy modification [19,20,21]. In recent times, natural substances including plant extracts have been the most popular modifiers of these polymers [22,23]. Particular attention has been paid to the use of *Equisetum arvense* L. (horsetail) which has shown pro-health activity [24]. *Equisetum arvense* L. has a very rich chemical composition. It consists of various compounds such as sterols (e.g., campesterol or isofucosterol), phenolic acids (e.g., caffeic acids or cinnamic acids), styryl-pyrones or flavonoids (e.g., quercetin or apigenin) [25]. According to literature reports, these compounds promote tissue repair and regeneration. For example, Goonoo and Bhaw–Luximon presented a role of flavonoids in tissue regeneration. They mentioned that flavonoids promote differentiation of pre-osteoblasts and mesenchymal stem cells into osteoblasts [26]. Additionally, *Equisetum arvense* L. contains some inorganic compounds, e.g., silicates, and it was proved that silica plays an important role in bone formation [27]. Results of numerous experiments show that *Equisetum arvense* L. extracts have an inductive effect on human osteoblasts while simultaneously inhibiting the activity of *Staphylococcus areus*. The potential application of horsetail extract for bone tissue regeneration was also verified by Arbabzadegan et al. They proved that the consumption of horsetail extract caused an increase in the mineral density of the mandibular bone [28]. In other work, it was proved that horsetail extract is recommended for patients suffering from diabetes because it promotes the regulation of blood glucose levels and the regulation of insulin resistance. Moreover, it was reported that this extract shows potential in the prevention of cardiomyopathy [29].

Despite many investigations confirming the pro-healthy activity of *Equisetum arvense* L. extract, no literature reports showing its application as a modifier of hydrogel dressings designed for chronic wound treatment were observed. Due to the unique properties of this extract, such as anti-inflammatory [30] or antibacterial activity [31], in addition to the fact that it may soothe pain and accelerate regenerative processes [32], this substance seems to be a promising modifier of such dressing materials. Thus, in this work, a synthesis methodology of hydrogel dressings based on chitosan and modified with *Equisetum arvense* L. extract is presented. The syntheses were performed with various amounts of crosslinking agent to verify which composition will provide materials with adequate mechanical properties. Next, in order to determine the possibility of wound exudate sorption by such dressings, studies on their sorption properties were conducted. The behavior of the hydrogels in simulated physiological liquids was verified, in addition to their physicochemical properties, including, e.g., wettability. Scanning electron microscopy (SEM) was used to characterize the surface morphology of hydrogels. Final experiments were aimed at determining the release profile of the active substance (plant extract) from developed hydrogels, while most attention was directed towards verifying in which environment (acidic or alkaline) this process was more effective.

## 2. Materials and Methods

### 2.1. Materials

Reagents used as a base for preparation of hydrogel materials such as chitosan (high molecular weight, Mw, 310–375 kDa; deacetylation degree 75–85%), crosslinking agent (diacrylate (PEGDA, poly(ethylene glycol)); average molecular weight Mn = 700 g/mol) and photoinitiator 2-hydroxy-2-methylpropiophenone (97%; d = 1.077 g/mL) were purchased from Sigma Aldrich (Saint Louis, MO, USA). *Equisetum arvense* L. (horsetail) extract in the form of dried leaves was bought in Herbamed Plus (Kraków, Poland).

### 2.2. Methods

#### 2.2.1. Preparation of *Equisetum arvense* L. Extract

The first part of the experiment included the preparation of the horsetail extract. For this purpose, 10 g of horsetail leaves were ground into a fine powder using a grinder. Then, water extraction was carried out, the powder was treated with water (100 mL), the solution was heated to 80 °C and remained for 15 min at this temperature. This procedure was repeated twice, while during the second time, the amount of water was reduced to 50 mL. Finally, the obtained extract was filtered using a corrugated filter, the filtrate obtained was centrifuged (2700 rpm; t = 15 min), and the supernatant was separated by decanting, and was lyophilized.

**Figure 1:**
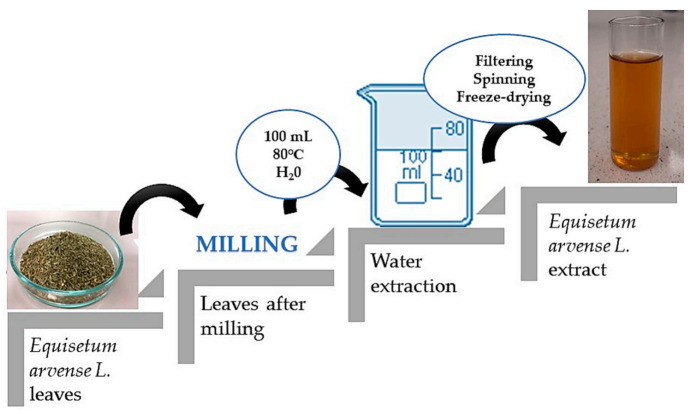
Scheme of preparation of horsetail extract.

In order to prepare horsetail extract, 2 g of prepared lyophilized powder was introduced into 50 mL of distilled water. The prepared suspension was boiled for 15 min. The scheme of the performed procedure is presented in Figure 1.

The obtained *Equisetum arvense* L. extract was used as a modifier of hydrogel materials in the remainder of the experiments.

#### 2.2.2. Synthesis of Hydrogels via a Photopolymerization Process

Syntheses of hydrogel materials were performed using UV radiation. Firstly, a base solution (i.e., 1% chitosan solution in 0.05% acetic acid solution) was mixed with appropriate amounts of crosslinking agent and photoinitiator. The whole reaction mixture was thoroughly mixed and introduced into the polymerization form (petri dish). Photopolymerization process aimed at the synthesis of hydrogels was carried out for 120 s while EMITA VP-60 (parameters: power, 180 W; λ = 320 nm) was used as a source of radiation.

Hydrogels modified with horsetail extract were prepared analogously. The only difference was that the reaction mixture was additionally supplemented with an adequate amount of this modifier. Detailed compositions of prepared hydrogels are presented in Table 1.

**Figure 2:**
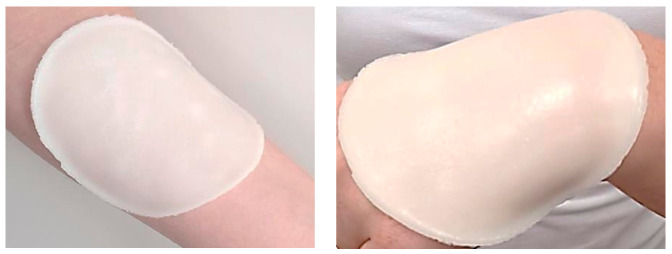
Example images of prepared hydrogel dressings (sample 12-10).

Below in Figure 2, example images of prepared hydrogels are presented.

Prepared hydrogels were subsequently investigated to evaluate their properties such as sorption capacity or tensile strength.

### 2.3. Methodology of Performed Experiments

#### 2.3.1. Characterization of Swelling Properties of Hydrogels

In view of the potential use of developed polymers as dressing materials, it was essential to determine their swelling ability. Innovative dressing should absorb wound exudate because its accumulation near the wound may inhibit the healing process, therefore, the main purpose of this research was to design a material which will provide an adequate environment for the wound healing process. Thus, to determine the swelling properties, hydrogel samples (weighing approximately 1.0 g) were immersed in selected liquids while the study was performed in SBF (simulated body fluid, isotonic to human blood plasma) and hemoglobin (porcine hemoglobin, used in a form of 2% aqueous solution). After selected time periods of 1 h, 24 h and 72 h, the swollen sample was separated from the solution and weighed. Swelling measurements were conducted at room temperature. Sorption ability of each sample was defined using swelling ratio (Q, g/g) calculated by means of the Equation (1):

(1) $$\alpha =\frac{\left(m-m_{0}\right)}{m_{0}}$$

where:

*α*—swelling ratio, g/g

*m*—mass of a swollen sample, g

*m*_0_—mass of a dry sample (before the analysis), g.

Results of the swelling studies were subsequently subjected to statistical analysis. The statistical significance was calculated by means of the two-way analysis of variance (ANOVA) (*α* = 5%). Analysis was performed to verify the importance of the swelling time and amount of the crosslinker. Measurements were conducted in three repetitions, from which an average value and the standard deviation (SD) were determined.

#### 2.3.2. Analysis of Surface Morphology of Hydrogels Using Scanning Electron Microscopy (SEM)

The surface morphology of hydrogels was characterized using SEM technique. The analysis was performed at room temperature. Hydrogels were firstly dried at room temperature and sputtered with gold. Imaging was conducted using the Jeol 5510LV scanning electron microscope (Jeol Ltd., Tokyo, Japan).

#### 2.3.3. Studies Aimed at Determining the Wettability of Hydrogels

The study involved defining the hydrophilicity of hydrogels via determining values of contact angles formed by the drop of distilled water on the surface of tested materials. The value of the contact angle and the shape of drop placed on the material’s surface depended strictly on the wetting properties of the analyzed samples. This study was conducted using Kruss DSA 100 M apparatus, and the geometric method was applied. Analyzed samples were placed on the stationary base and treated with a drop of distilled water provided via a micropipette. The behavior of the drop of the measuring liquid in contact with tested samples was recorded using an optical system equipped with a digital camera. For each hydrogel sample, six measurements (three from the top surface and three from the bottom surface) were performed, and the results are presented as an average value of the measurements and the standard deviation (SD).

#### 2.3.4. Behavior of Hydrogels in an Environment of Simulated Physiological Liquids

Investigations were performed to verify the possible interactions between simulated physiological liquid and an immersed hydrogel sample. Incubation was carried out in the same liquids as the swelling studies, and, importantly, in distilled water as a reference liquid. The study involved the immersion of hydrogel samples (weight approximately 1.0 g) in 50 mL of selected liquid. Next, the systems were introduced into the laboratory incubator to provide temperature conditions simulating human body temperature, i.e., 36.6 °C. Incubation studies were carried out for 12 days, and the pH of the incubation liquid was measured every day.

#### 2.3.5. Investigations on the Release of Active Substance from the Interior of Hydrogel Materials

Hydrogels were designed for application as innovative dressing materials which absorb the wound exudate and at the same time release an active substance to the wound environment. Thus, this study was conducted to verify the possibility of such a release from the developed materials, and—if it took place—to verify the conditions in which this process was more effective. For this purpose, the study was performed in two environments, i.e., at pH = 2.0 (2% solution of citric acid) and at pH = 7.4 (phosphate buffer). The first task was to select a constituent compound of horsetail extract whose presence in tested solution could be measured spectrophotometrically. Among many compounds, saccharides were selected, as their reaction with aluminum chloride (AlCl_3_) forms colorful compounds which may be detected via UV-Vis spectrophotometry.

The analysis was performed at 36.6 °C (the same as in the incubation studies, which was aimed at simulating human body conditions). Firstly, hydrogel samples were introduced into flasks containing 200 mL of tested environments (phosphate buffer and 2% solution of citric acid), placed in the shaking incubator (Hanchen ES-60E temperature controlled incubator and Shaker Scientific Incu-Shaker shaking incubator) and were shaken (shaking speed = 80 rpm). Next, after specific time periods, 3 mL of the tested solutions were taken to the cuvettes and mixed with 0.125 mL of 3% AlCl_3_ methanolic solution to initiate the reaction of obtaining colorful complexes. After 30 min, the mixture in the cuvette was analyzed spectrophotometrically by the application of the UV–Vis visible spectrophotometer V-500 apparatus, and the maximum absorbance was observed at 310 nm. Importantly, the volume of flasks after each sampling was supplemented to the initial volume.

#### 2.3.6. Assessment of Mechanical Properties of Hydrogels

Mechanical properties of the prepared polymers were evaluated in accordance with ISO 527-2 type 5A and ISO 37 type 2 standards. Firstly, paddle-shaped hydrogel samples were prepared by means of ZCP020 blanking die and dried under pressure (to keep their shapes) at room temperature for 24 h. Prepared “paddles” were next placed in the jaws of the apparatus while the analysis was performed using Brookfield CT3 (AMETEK Brookfield, Chandler, AZ, USA) texture analyzer. The analysis allowed the determination of the tensile strength (***R_m_***) of each sample using Equation (2), and their elongation (***A***) using Equation (3). Both equations are presented below.

(2) $$R_{m}\;=\frac{F_{m}}{S_{0}}$$

(3) $$A=\frac{100\;\cdot \;\left(l_{u}-l_{0}\right)}{l_{0}}$$

*F_m_*—maximum strength of tested sample

*S*_0_—cross-sectional area of the sample in its initial state

*l_u_*—measured length after sample rupture

*l*_0_—measured length of the sample at the beginning of the analysis.

## 3. Results and Discussion

### 3.1. Results of Swelling Investigations

**Figure 3:**
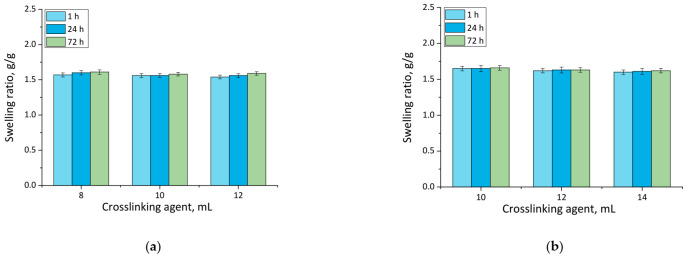
Swelling in SBF of unmodified hydrogels (**a**), and hydrogels modified with 10 mL of *Equisetum arvense* L. extract (**b**) (number of repetitions *n* = 3).

**Figure 4:**
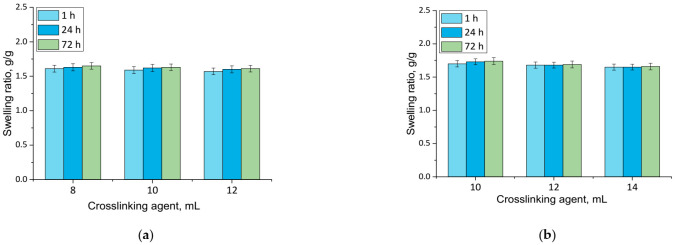
Swelling in hemoglobin of unmodified hydrogels (**a**) and hydrogels modified with 10 mL of *Equisetum arvense* L. extract (**b**) (number of repetitions *n* = 3).

Results of performed analyses are presented in a form of bar charts providing information on swelling properties of analyzed samples in addition to the impact of the modifier on these properties. Values of calculated swelling ratios (Q, g/g) of hydrogel materials are shown in Figure 3 (swelling in SBF) and Figure 4 (swelling in hemoglobin) while results of performed statistical analysis are presented in Table 2 and Table 3.

Firstly, the experiments allowed the conclusion that all prepared hydrogels showed swelling properties. Nonetheless, both unmodified hydrogels and materials containing horsetail extract exhibited slightly different swelling ratios depending on the amount of crosslinking agent used during the synthesis. The greater the amount of crosslinker used, the lower the swelling capability of tested material. This, in turn, resulted from the fact that the amount of crosslinker used affected the crosslinking degree of the hydrogel, i.e., the greater the amount of this reagent, the higher the crosslinking density. As a result, such crosslinked material showed fewer free spaces in its polymer network available for absorbed liquid, and hence this material had a lower swelling ratio. Similar conclusions concerning the dependance between the swelling ratio and the crosslinking density were drawn in studies presented in [33,34,35,36,37].

Moreover, it was proved that there was a slight difference between swelling ability of unmodified hydrogels and hydrogels containing horsetail extract, i.e., modified materials swelled slightly more. This may have been caused by the release of the plant extract from the hydrogel material being incubated. The horsetail extract release contributed, in turn, to the increase in the number of free spaces between polymer chains in polymer networks, which finally resulted in a possibility of penetration of these free spaces—occupied previously by the modifier—by absorbed liquid.

### 3.2. Evaluation of the Surface Morphology of Hydrogels

**Figure 5:**
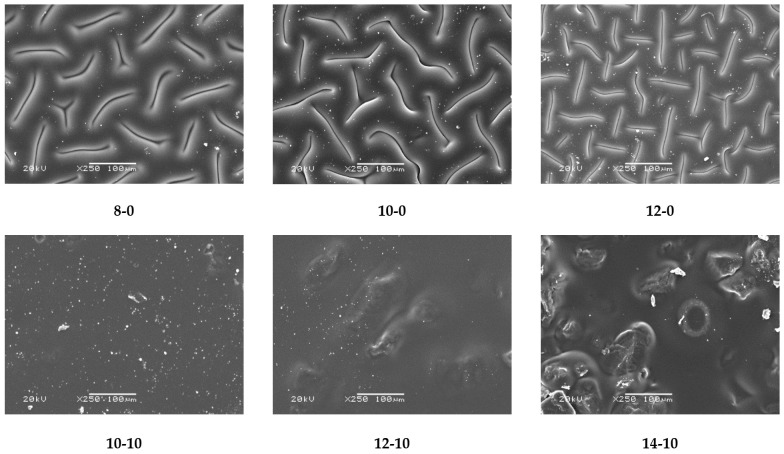
SEM images of obtained hydrogel materials.

The next analysis involved determining the impact of the modifying agent and the amount of the crosslinking agent used during the synthesis on the surface morphology of obtained hydrogels. Images prepared via a SEM technique are presented in Figure 5.

Obtained SEM images allowed the observation that in the case of both unmodified and modified hydrogel materials, an increase in the amount of crosslinking agent used in the synthesis caused an increase in the crosslinking density of the polymer matrix and hence also the considerable surface corrugation. Moreover, a clear impact of the modifier on the hydrogels’ surface morphology was also observed. Modified hydrogels were characterized by a significantly smoother surface, compared with the surface of unmodified samples. It is probable that the horsetail extract filled the free spaces between polymer chains forming the polymer matrix, and this was reflected in a smoother surface.

### 3.3. Analysis of the Wettability of Hydrogels

**Figure 6:**
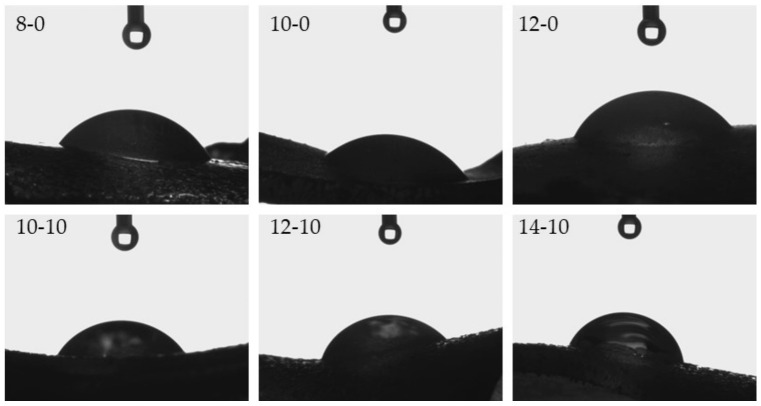
Images presenting the wettability of hydrogels towards distilled water.

One of the key aspects in tissue regeneration is the phenomenon of protein adsorption and cell adhesion at the site of damaged tissue. Both of these processes affect cell proliferation, and, as a result, tissue regeneration [38]. The spread and proliferation of fibroblasts, i.e., cells of connective tissue occurring in the dermis, depend strongly on the surface properties of applied biomaterials [39]. Therefore, it is very important to determine the hydrophilic or hydrophobic nature of the surfaces of prepared hydrogels with potential use as innovative wound dressing materials, and hence investigation of wettability was performed. Images from the performed experiments are presented in Figure 6, while the mean values from six measurements together with the standard deviations are shown in Table 4.

Considering the results presented above, it may be observed that hydrogels modified with horsetail extract showed significantly more hydrophilic surface than unmodified materials. Contact angles of hydrogels containing *Equisetum arvense* L. extract were also greater, compared with contact angles of polymers without this additive. This may be due to the presence of hydrophilic groups derived from the modifying agent increasing the surface affinity to water. Furthermore, it may be noticed that as the amount of crosslinking agent used in the synthesis increased, a bigger value of the contact angle of the obtained hydrogel was obtained. This was due to the previously described increase in the crosslinking degree of the polymer structure. Such an increase may have caused a greater corrugation of the polymer structure via a higher packing density of polymer chains. This, in turn, was also confirmed by means of SEM technique, the results of which were discussed in a previous subsection. Moreover, it may be assumed that the very densely packed polymer chains caused the droplets to be retained in the pores of the material, therefore, in the case of the greater amount of the crosslinking agent, a bigger value of contact angle was observed. Importantly, another reason may have been the occurrence of the more frequent presence of side-by-side functional groups, due to the greater crosslinking density. A larger number of functional groups on the surface of the material resulted in the drop location on this surface, due to physical interactions such as hydrogen bonds. As a result, a drop of distilled water was held in place and did not spread over the surface of the material, and consequently the contact angle was greater for materials prepared using a greater amount of crosslinking agent.

### 3.4. Results of Incubation Studies in Simulated Physiological Liquids

**Figure 7:**
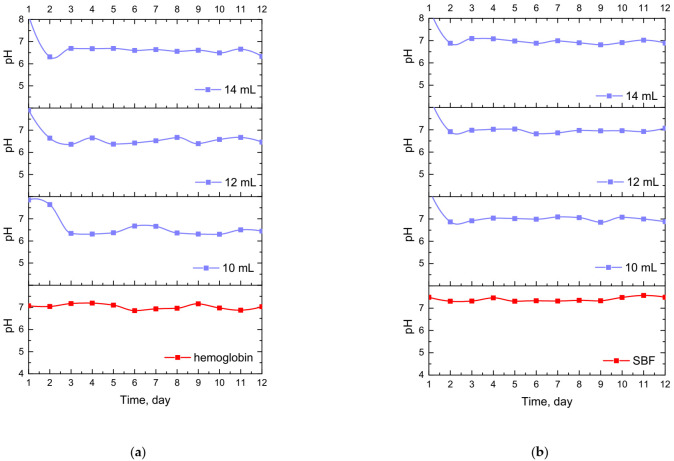

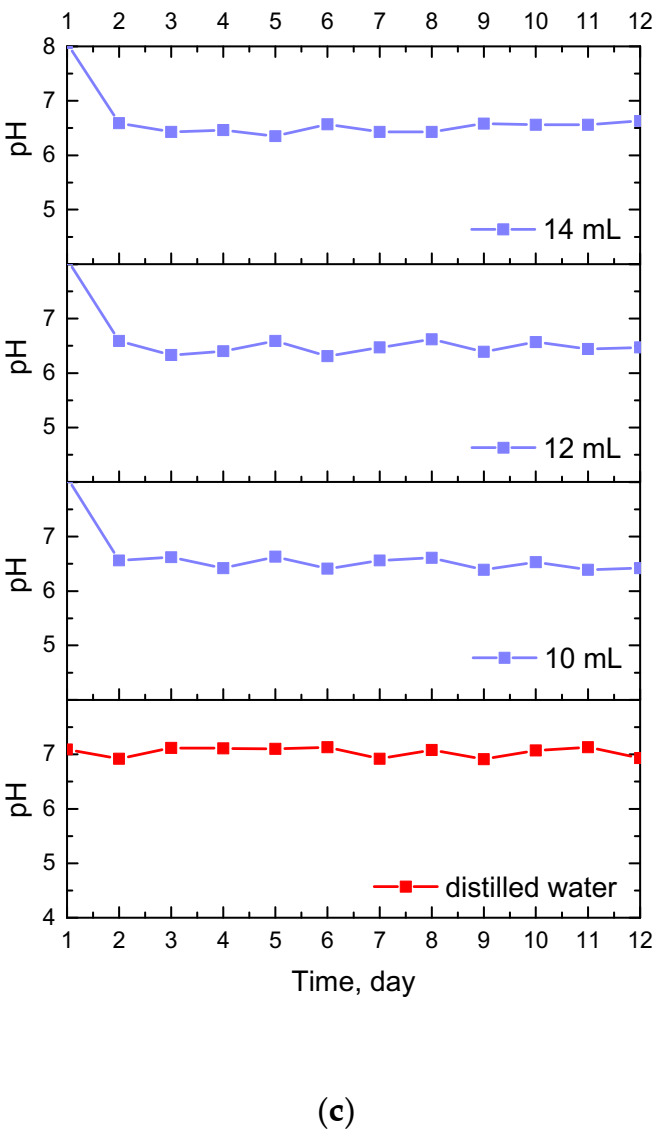
Changes in pH values determined during incubation of hydrogels modified with *Equisetum arvense* L. extract in hemoglobin (**a**), SBF (**b**) and distilled water (**c**).

Results of the incubation of hydrogel materials performed in SBF, hemoglobin and distilled water are presented below in Figure 7.

The study allowed the determination of the impact of hydrogel materials on pH values of liquids in which they were incubated. Based on the research, it was possible to observe a slight change in the pH of the liquids at the beginning of the incubation, then pH values stabilized, and were maintained until the end of the measuring period. According to the literature data, 1% horsetail extract shows pH = 6.2 [40]. Thus, the initial change in pH (i.e., a slight decrease) mentioned previously probably resulted from the release of this additive from the hydrogel materials to the tested media. This, in turn, was related to the slight acidification of the media in which incubation took place. Nonetheless, buffering properties of hydrogels caused the pH values to stabilize in the latter part of the measurements.

### 3.5. Investigations on the Release of Active Substance from Hydrogel Matrices

**Figure 8:**
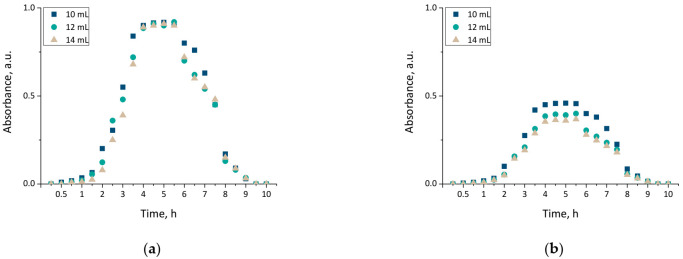
The release profiles of materials modified with *Equisetum arvense* L. extract determined in 2% citric acid solution (**a**) and phosphate buffer (**b**).

Modification of hydrogel material via an introduction of various substances into their matrices results in an enhancement of the material with new properties, including therapeutic properties, and thus, increasing the spectrum of their potential application. Nonetheless, a key aspect of this investigation was to verify whether this substance was released from the interior of the modified hydrogel. The release of horsetail extract was checked in two environments, i.e., acidic and alkaline, and verified via UV-Vis spectrophotometry. Obtained results are presented in Figure 8.

The results presented above allowed the conclusion that the release of horsetail extract from developed hydrogels was significantly more efficient in an acidic environment than in an alkaline one. This was probably caused by the phenomenon of protonation of NH_2_ groups via hydrogen ions. These groups derived from chitosan, while the hydrogen ions derived from citric acid forming acidic environment. As a result of these interactions NH_3_^+^ ions were formed. Electrostatic repulsion between these ions resulted, in turn, in an increase in the distance between polymer chains, and simultaneously caused a release of modifier from these spaces. Therefore, the release process proceeded more efficiently than when such polymer chains were packed more densely. Thus, in an alkaline environment, the release of active substance also occurred but was less effective, i.e., only 50% of the horsetail extract was released over the same time. Similar conclusions concerning more efficient drug release from chitosan hydrogels in acidic environment, than in an alkaline environment, were also drawn by Wu et al. [41].

The results of the experiments indicated that the research direction chosen in this work was proper, and confirmed a potential for designed hydrogels to be applicable as wound dressing materials with the function of a release of an active substance (in this case, horsetail extract).

### 3.6. Analysis of Mechanical Properties of Hydrogels

**Figure 9:**
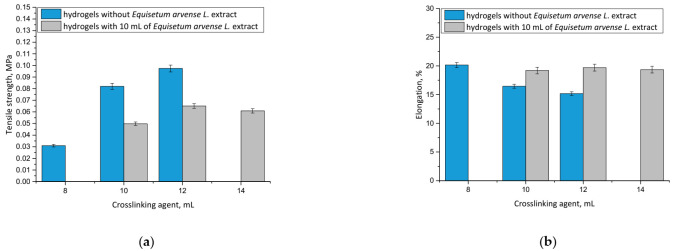
Results of mechanical analyses of both unmodified hydrogels and hydrogels containing horsetail extract prepared using various amounts of crosslinking agent showing values of the tensile strength (**a**), and percentage elongation (**b**) (*n* = 3, *n*—number of repetitions).

Considering the potential application of hydrogels as wound materials, it was also important to evaluate their mechanical properties. Dressing materials need to be characterized by an adequate tensile strength, and simultaneously provide an elasticity and ease of application to the wound, which is why these experiments were performed. The results, including the properties of both unmodified and modified hydrogels, are presented in Figure 9.

It may be noticed that hydrogel materials modified with plant extract showed greater elasticity than unmodified polymers. For example, the hydrogel sample prepared using 10 mL of crosslinking agent and 10 mL of horsetail extract was characterized by a percentage elongation of A = 19.20% while the unmodified sample obtained using the same amount of crosslinker showed A = 16.45%. This was probably caused by the fact that an introduction of 10 mL of the modifying agent into the reaction mixture resulted in its dilution, while simultaneously the same amount of crosslinking agent was added as in the unmodified hydrogel. As a result, the obtained material showed a lower crosslinking degree, and therefore, a less compact structure, which, in turn, translated into its greater elasticity.

Conversely, it was proved that by using a specific amount of the crosslinking agent in the synthesis, it was possible to obtain material with a desirable elasticity, depending on its application site. The greater the amount of crosslinking agent applied, the more the hydrogel structure was crosslinked. However, it should be noted that too large an amount of this reagent may negatively affect the mechanical properties of the final material. This was observed, for example, in the case of the modified hydrogel sample obtained using 14 mL of the crosslinker, which showed lower elasticity than modified hydrogel prepared with 12 mL of the crosslinker. This, in turn, indicated that too large an amount of this reagent was applied, and that material’s elasticity was lower, as it was observed to be tough and stiff.

It may be also stated that with an increasing amount of crosslinking agent, an increase in the tensile strength of the hydrogel was observed. This was also reported by Wong et al. [42], Haryanto et al. [43] and Lai et al. [44].

The most important priority of this work was to determine the mechanical properties of each prepared sample and discuss the impact of both the amount of the modifier and the crosslinker on its elasticity and tensile strength. Based on these investigations it may be stated that materials containing horsetail extract showed better therapeutic properties (due to the large amount of the additive) and better tensile strength compared with unmodified samples prepared with the same amount of crosslinker. Therefore, these dressing materials will provide more active substances to the wound, but they may be placed in a site with lower mobility (due to their slightly lower elongation) compared with unmodified samples containing the same amount of PEGDA. It is important to discuss the properties of prepared hydrogels depending on the location and properties of the wound. For example, more elastic samples may be used for wounds located in places with higher mobility, therefore, such materials may be applied for a larger spectrum of wounds.

## 4. Conclusions

- Developed hydrogels showed swelling properties while their modification with horsetail extract resulted in an increase in their swelling ability;
- The larger the amount of crosslinker used in the synthesis of hydrogels, the lower their swelling capacity. This, in turn, was related to the greater crosslinking density of materials obtained with the larger amount of the crosslinker;
- The increase in the crosslinking density of hydrogels was also reflected in their surface morphology. The higher the crosslinking density, the greater the surface corrugation;
- Hydrogels modified with horsetail extract showed smoother surface than unmodified hydrogels;
- All prepared hydrogels showed hydrophilic nature—contact angles determined for all measured samples were lower than 77°. For hydrogels modified with horsetail extract, lower contact angles (therefore, higher hydrophilicity) were determined than for unmodified samples;
- During incubation of hydrogels in simulated physiological liquids, an initial slight acidification of incubation media was observed, which probably resulted from the release of the horsetail extract from polymer matrices to the tested environments. However, in the course of further measurements, the pH of the liquids stabilized which was, in turn, related to the buffering properties of hydrogels;
- The release of horsetail extract from hydrogel matrices was twice as effective in an acidic environment than in an alkaline one;
- By selecting adequate amounts of both crosslinking agent and modifying agent, it was possible to obtain materials with adequate mechanical properties providing, at the same time, therapeutic activity;
- Developed hydrogel materials modified with horsetail extract showed swelling ability, which provided wound exudate sorption. Furthermore, these materials showed hydrophilic nature which promoted cell proliferation, and were characterized by adequate tensile strength with simultaneous elasticity. Thus, it was assumed that such designed hydrogels show great application potential and are appropriate to be subjected to more advanced studies including, e.g., detailed biological analysis.

## Figures and Tables

**Table 1:** Compositions of hydrogels modified with *Equisetum arvense* L. extract.

Base Solution, mL	Photoinitiator, mL ^a^	Crosslinking Agent, mL ^b^	*Equisetum arvense* L. Extract, mL	Sample Name
50.0	0.5	8.0	-	8-0
		10.0		10-0
		12.0		12-0
50.0	0.5	8.0 *	10.0	-
		10.0		10-10
		12.0		12-10
		14.0		14-10

^a^ 2-hydroxy-2-methylpropiophenone, Darocur 1173, ^b^ diacrylate poly(ethylene glycol), * sample was not crosslinked properly thus it was not considered for further investigations.

**Table 2:** Statistical analysis of swelling studies in SBF based on the two-way analysis of variance (ANOVA) with repetitions.

Independent Variable	Sample	* *p*
Amount of crosslinking agent	modified	0.06988
Time	modified	0.02780
Amount of crosslinking agent	unmodified	0.40404
Time	unmodified	0.04495

* *p* indicates the statistical significance calculated using the two-way analysis of variance (ANOVA).

**Table 3:** Statistical analysis of swelling studies in hemoglobin based on the two-way analysis of variance (ANOVA) with repetitions.

Independent Variable	Sample	* *p*
Amount of crosslinking agent	modified	0.17837
Time	modified	0.02440
Amount of crosslinking agent	unmodified	0.09313
Time	unmodified	0.03854

* *p* indicates the statistical significance calculated using the two-way analysis of variance (ANOVA).

**Table 4:** Values of contact angles of tested hydrogels.

Sample Name	Contact Angle ± SD, °
8-0	68.80 ± 1.15
10-0	72.60 ± 1.07
12-0	74.55 ± 1.05
10-10	54.95 ± 0.98
12-10	69.00 ± 1.85
14-10	76.50 ± 1.50

## Data Availability

The data presented in this study are available on request from the corresponding authors.
